# Current status of wearable cardioverter‐defibrillator use in Japan

**DOI:** 10.1002/joa3.13113

**Published:** 2024-07-14

**Authors:** Reina Tonegawa‐Kuji, Taku Nishida, Yoko Sumita, Yoshihiro Miyamoto, Koshiro Kanaoka

**Affiliations:** ^1^ Department of Medical and Health Information Management National Cerebral and Cardiovascular Center Suita Japan; ^2^ Department of Cardiovascular Medicine Nara Medical University Nara Japan; ^3^ Open Innovation Center National Cerebral and Cardiovascular Center Suita Japan

**Keywords:** arrhythmia, cardiopulmonary arrest, implantable cardioverter‐defibrillator, sudden cardiac death

## Abstract

**Background:**

The current status of wearable cardiovascular defibrillators (WCD) use in Japan is unclear.

**Methods:**

Using a nationwide claims database of Japan, we assessed characteristics of patients using WCD and factors influencing subsequent implantable cardioverter‐defibrillator (ICD) implantation.

**Results:**

In 1049 cases, those with prior cardiopulmonary arrest (CPA) or ventricular arrhythmia, cardiomyopathy, or device‐related issues were more likely to require permanent ICDs, whereas females were less likely.

**Conclusions:**

Prior CPA or fatal arrhythmia, underlying cardiomyopathy, or device‐related issues were associated with future permanent ICD implantation. These findings offer insights into the current status of WCD use in Japan.

## Graphical abstract

Using a nationwide claims database of Japan, we assessed characteristics of patients using wearable cardiovascular defibrillators and factors influencing subsequent implantable cardioverter‐defibrillator (ICD) implantation. In 1049 cases, those with prior cardiopulmonary arrest or ventricular arrhythmia, cardiomyopathy, or device‐related issues were more likely to require permanent ICDs, whereas females were less likely.

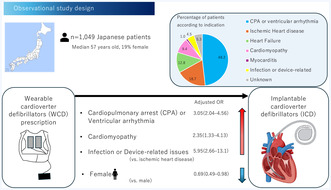

A wearable cardiovascular defibrillator (WCD) is used for patients at risk of sudden cardiac death. Although several reports on WCDs exist, including one randomized controlled trial from Western countries, reports on WCDs from Japan are limited mainly to small cohort studies.^1^, ^2^ This study aimed to investigate patient characteristics and the rates of permanent implantable cardioverter‐defibrillator (ICD) implantation following WCD use in Japan. The study protocol was approved by the Institutional Review Board of Nara Medical University (approval no. 3021). We used the Japanese Registry of All Cardiac and Vascular Diseases‐Diagnosis Procedure Combination (JROAD‐DPC), a nationwide inpatient claims database.^3^ Between April 2014 and March 2020, 1222 hospitalizations with WCD prescriptions were identified, with 173 excluded because of a lack of a 3‐month follow‐up. This resulted in 1049 hospitalizations with WCD prescriptions being analyzed (median age, 57 years [IQR: 44–68]; 19% female) (Table 1). Patients were grouped based on underlying cardiac disease type, as per the International Classification of Diseases 10th revision codes in the main, the admission‐precipitating, and the most‐resource‐consuming diagnosis. Mixed‐effects, multivariable logistic regression analysis using institute as a random intercept, with adjustment for age, gender, and comorbidities (hypertension, diabetes, and dyslipidemia) was performed to investigate the association between underlying cardiac disease and ICD implantation within the same fiscal year. A total of 581 (55.4%) patients underwent ICD implantation within the same fiscal year. ICD implantation after WCD use was relatively high among patients with cardiopulmonary arrest (CPA) or ventricular arrhythmia, and in those with infection‐ or device‐related problems (64.8% and 76.6%, respectively). The time from WCD implementation to ICD implantation was shorter among patients with CPA or ventricular arrhythmia compared with those without (19 [9–40] vs. 44 [20–74] days, *p* < .001). Conversely, the time from WCD implementation to ICD implantation was longer in patients with ischemic heart disease than those without (51 [25–75] vs. 24 [10–56] days, *p* < .001). History of CPA or ventricular arrhythmia (odds ratio [OR], 3.05 [2.04–4.56], *p* < .001), cardiomyopathy (OR, 2.35 [1.33–4.13], *p* = .003), and infection or device‐related problems (OR, 5.95 [2.66–13.3], *p* < .001) had a higher probability of requiring permanent ICD implantations in the future (reference: ischemic heart disease). Gender‐based differences were also observed, with females being inversely associated with the future decision for permanent ICD implantation (OR, 0.69 [0.49–0.98], *p* = .04). This study has some limitations. Some important information was not available, including WCD usage duration, especially for those without ICD implantation, as well as information on death or unexpected rehospitalization among WCD users, and whether ICDs were implanted for primary or secondary prevention. Additionally, while some patients eligible for an ICD may have opted out because of personal preferences, the rate of such declinations was not known, potentially affecting the analysis outcomes. Owing to many unknown cases of underlying heart disease, the primary diagnosis was prioritized. It is believed subsequent outpatient examinations may have clarified diagnoses for some; however, only information from hospitalization was accessible. Finally, few patients with myocarditis were included, making it difficult to capture a clear trend.

**TABLE 1:** Patient characteristics.

	Total	CPA or ventricular arrhythmia	Ischemic heart diseases	Heart failure	Cardiomyopathy	Myocarditis	Infection‐ or device‐related	Unknown
	*N* = 1049	*N* = 506	*N* = 196	*N* = 134	*N* = 99	*N* = 11	*N* = 47	*N* = 56
Age (years)	57 (44–68)	53 (40–67)	63 (53–71)	59 (48–68)	54 (37–63)	49 (28–55)	54 (43–68)	57 (44–68)
Women	185 (18%)	84 (17%)	22 (11%)	30 (22%)	25 (25%)	4 (36%)	11 (23%)	9 (16%)
Emergency	653 (62%)	308 (61%)	156 (80%)	89 (66%)	43 (43%)	10 (91%)	24 (51%)	23 (41%)
Hypertension	411 (39%)	163 (32%)	115 (59%)	65 (49%)	28 (28%)	3 (27%)	13 (28%)	24 (43%)
Diabetes	309 (29%)	108 (21%)	101 (52%)	48 (36%)	19 (19%)	5 (45%)	14 (30%)	14 (25%)
Dyslipidemia	480 (46%)	189 (37%)	178 (91%)	56 (42%)	25 (25%)	1 (9%)	14 (30%)	17 (30%)
PCI	197 (19%)	37 (7%)	138 (70%)	16 (12%)	1 (1%)	0 (0%)	0 (0%)	5 (9%)
CABG	38 (3.6%)	2 (0.4%)	30 (15.3%)	6 (4.5%)	0 (0%)	0 (0%)	0 (0%)	0 (0%)
RFCA	117 (11%)	75 (15%)	2 (1%)	13 (10%)	4 (4%)	0 (0%)	1 (2%)	22 (39%)
IABP	101 (10%)	16 (3%)	58 (30%)	18 (13%)	3 (3%)	2 (18%)	0 (0%)	4 (7%)
PCPS	28 (2.7%)	10 (2.0%)	6 (3.1%)	4 (3.0%)	1 (1.0%)	5 (45.5%)	0 (0.0%)	2 (3.6%)
Length of hospital stay (days)	22 (13–34)	17 (10–27)	26 (17–37)	27 (21–46)	23 (12–48)	38 (21–100)	30 (13–50)	22 (7–32)
ICD implantation after WCD use	581 (55.4%)	328 (64.8%)	83 (42.3%)	51 (38.1%)	55 (55.6%)	5 (45.5%)	36 (76.6%)	23 (41.1%)
Time WCD implementation to ICD implantation (days)	27 (11–61)	19 (9–40)	51 (25–75)	54 (18–84)	31 (9–64)	75 (27–82)	41 (19–69)	44 (14–85)

Abbreviations: CABG, coronary artery bypass surgery; CPA, cardiopulmonary arrest; IABP: Intra‐aortic balloon pump; PCI, percutaneous coronary intervention; PCPS, percutaneous cardiopulmonary support; RFCA, radiofrequency catheter ablation.

Herein, we identified the characteristics of patients with WCDs and clinical factors relevant to subsequent ICD implantation using a real‐world database. The effectiveness of the WCD remains controversial,^2^ and more detailed analyses regarding its efficacy are warranted in future studies.

## FUNDING INFORMATION

This work was supported by JSPS KAKENHI (Grant number JP22H03365).

## CONFLICT OF INTEREST STATEMENT

Authors declare no conflict of interests for this article.

## ETHICS STATEMENT

This study was approved by the Institutional Review Board of Nara Medical University (Approval no. 3021).

## RELATIONSHIP WITH INDUSTRY

No industry relationships to declare.

## PATIENT CONSENT STATEMENT

N/A.

## CLINICAL TRIAL REGISTRATION

N/A.

## Data Availability

The data that support the findings of this study are available upon reasonable request.
